# Thiopental Versus Propofol in Combination with Remifentanil for Successful Classic Laryngeal Mask Airway Insertion: A Prospective, Randomised, Double-Blind Trial †

**DOI:** 10.3390/ph18081173

**Published:** 2025-08-08

**Authors:** Mert Akan, Mensure Çakırgöz, İsmail Demirel, Ömürhan Saraç, Aysun Afife Kar, Ergin Alaygut, Oğuzhan Demirel, Hicret Yeniay, Abdurrahman Tünay

**Affiliations:** 1 Intensive Care Unit, Department of Anesthesiology and Reanimation, Izmir Acibadem Kent Hospital, 35580 Izmir, Turkey; 2 Intensive Care Unit, Department of Anesthesiology and Reanimation, Izmir City Hospital, University of Health Sciences, 35120 Izmir, Turkey; drmensure@gmail.com (M.Ç.); dromurhansarac@hotmail.com (Ö.S.); aaysunkar@hotmail.com (A.A.K.); hicret.yeniay@yahoo.com (H.Y.); 3 Intensive Care Unit, Department of Anesthesiology and Reanimation, Firat University School of Medicine Hospital, 23119 Elazig, Turkey; ismaildemirel23@gmail.com; 4 Intensive Care Unit, Department of Anesthesiology and Reanimation, Izmir Tepecik Training and Research Hospital, University of Health Sciences, 35180 Izmir, Turkey; ealaygut@yahoo.com; 5 Department of Anesthesiology and Reanimation, Ankara University Ibni Sina Hospital, 06230 Ankara, Turkey; demireloguzhan1@gmail.com; 6 Department of Anesthesiology and Reanimation, Istanbul Training and Research Hospital, 34098 Istanbul, Turkey; atunay.02@hotmail.com

**Keywords:** remifentanil, propofol, thiopental, laryngeal mask airway

## Abstract

**Background**: Remifentanil, an ultra-short-acting μ-receptor agonist, is used with propofol or thiopental for tracheal intubation without muscle relaxants. While effective with both, its combination with thiopental provides better hemodynamic stability. Thiopental has long been a standard intravenous agent for anaesthesia induction and remains a cost-effective alternative to propofol in resource-limited settings. To date, no study has directly compared the effects of thiopental–remifentanil and propofol–remifentanil combinations on LMA insertion conditions. This study aims to compare the effects of thiopental or propofol with 2 µg·kg^−1^ remifentanil on laryngeal mask airway (LMA) insertion conditions and success in a prospective, randomised double-blind study. **Method**: The study included 80 premedicated ASA I-II patients, aged 18–65, randomised into Group P (propofol) and Group T (thiopental). Anaesthesia induction was with 2 μg·kg^−1^ remifentanil, followed by 5 mg·kg^−1^ thiopental or 2.5 mg·kg^−1^ propofol. LMA insertion occurred 90 s post-induction. LMA insertion conditions were evaluated using a six-variable scale. Systolic arterial pressure (SAP), diastolic arterial pressure (DAP), mean arterial pressure (MAP), heart rate (HR), and bispectral index monitor (BIS) values were recorded at baseline, 1 min pre-insertion, and at 1, 2, 3, 4, and 5 min after insertion. Apnoea duration, loss of eyelash reflex duration, insertion duration, number of attempts, and perioperative complications were also documented. **Results**: Demographic data were similar. Group P showed significantly shorter eyelash reflex loss and LMA insertion durations, longer apnoea duration, and higher rates of full mouth opening, excellent LMA insertion condition, and hypotension or bradycardia compared to Group T (*p* < 0.05). Group P had significantly lower HR, SAP, DAP, and MAP at various time points (*p* < 0.05). There were no significant differences in blood presence on LMA, sore throat, or dysphagia (*p* > 0.05). **Conclusions**: In our study, administration of 2 μg·kg^−1^ remifentanil before induction along with thiopental or propofol was shown to provide acceptable LMA insertion conditions at comparable levels. As hemodynamic parameters were less affected, we believe the remifentanil–thiopental combination may be a suitable alternative.

## 1. Introduction

Airway management is a basic and important skill in anaesthesia practice. The non-invasive supraglottic airway device (SAD), known as laryngeal mask airway (LMA), is a reliable and simple technique for anaesthesia [1]. It has been used in clinical practice since the late 1980s, and has been recommended for emergency airway by the American Society of Anesthesiologists since 1993 [1,2].

LMA insertion may be performed blind without a laryngoscope; as a result, it is less invasive than endotracheal intubation and may be completed without muscle relaxants [1,3]. However, to be able to insert LMA, a sufficient mouth opening should be provided, and there should be sufficient anaesthesia depth to suppress reflexes like coughing, retching, or laryngospasm in the upper airway [3,4,5,6]. With this aim, several studies have been performed on the agents and induction techniques, providing optimum conditions. In previous studies, it was reported that LMA insertion duration with intravenous anaesthetics was shorter compared to inhalation anaesthetics, and patient satisfaction was higher. Because of its superior ability to attenuate laryngeal responses and upper airway reflexes compared with thiopental, propofol has emerged as the most commonly preferred agent for LMA insertion [5,6,7,8]. However, administration of propofol as a sole agent in non-premedicated patients necessitates a high bolus dose (2.5–3.0 mg·kg^−1^) or elevated target plasma concentration (7–9 ng·mL^−1^), which can potentially induce significant cardiovascular and/or respiratory depression [9]. Additionally, its formulation in a lipid emulsion increases the risk of microbial contamination, causes significant pain at the injection site, and leads to increased costs, thereby limiting its use in developing countries [3,10,11,12]. Much research has been conducted on cheaper and equally effective induction techniques; accordingly, thiopental is among the most frequently selected agents [5,6,8,10,11,13,14,15,16,17,18]. Introduced into clinical practice in 1934, thiopental was, for many years, the most widely used intravenous hypnotic agent [19]. Although its global use has declined, regional differences persist; following the cessation of its production in 2011, it has been almost entirely abandoned in the United States, while in the United Kingdom and Europe, it continues to be used in limited settings such as obstetric cases, electroconvulsive therapy, and neuroanaesthesia [19,20,21]. Nevertheless, due to its rapid onset, short duration of action, and cardiovascular stability, it remains widely preferred in developing countries, particularly where access to propofol is limited [14,15,16,18]. However, when standard induction doses are administered in isolation, optimal conditions for successful LMA placement cannot be achieved [8,10,22]. As a result, studies performed about induction techniques providing LMA insertion conditions at comparable rates to propofol without increases in apnoea duration and cardiovascular depression have used thiopental along with several agents as adjuvants [3,5,6,8,10,13,22].

Remifentanil, a 4-anilidopiperidine derivative of fentanyl, is a μ-opioid receptor agonist characterised by an ultra-short duration of action. The effect begins rapidly (1 min) and ends rapidly (half-life less than 10 min) [23]. The combination of propofol with remifentanil was reported to improve the success rate of excellent or clinically acceptable conditions for LMA insertion and intubation without the use of muscle relaxants, while not markedly prolonging apnoea duration, compared to propofol alone or in combination with other opioids; however, the risk of hypotension and bradycardia increased with escalating doses [23,24,25,26,27,28,29]. Durmuş et al. [30] and Mohammadreza S et al. [31] reported that 4 μg·kg^−1^ remifentanil before induction with 5 mg·kg^−1^ thiopental provided excellent and satisfactory intubation conditions at 95% rates without muscle relaxants, with acceptable levels of hemodynamic suppression without causing increases in hypotension and bradycardia risk. However, based on the sources we have access to, no study has been found that both evaluates the effect of the combination of remifentanil and thiopental on LMA insertion conditions and compares it to the effect on LMA insertion conditions when administered with propofol.

Despite its global decline, thiopental remains a useful alternative to propofol in resource-limited settings where access to propofol is restricted. Accordingly, this prospective, randomised, and double-blind clinical study aimed to compare the effects of 2.5 mg·kg^−1^ propofol and 5 mg·kg^−1^ thiopental, each combined with 2 μg·kg^−1^ remifentanil administered prior to induction, on hemodynamic changes and laryngeal mask airway (LMA) insertion conditions in patients undergoing general anaesthesia for surgeries lasting less than two hours and not requiring tracheal intubation.

## 2. Results

The demographic data, including age, gender, weight, and the duration of LMA application, showed no notable differences between the two groups (Table 1). The duration to loss of eyelash reflex and the LMA insertion duration were significantly shorter in Group P compared to Group T (*p* < 0.001). The apnoea duration was significantly longer in Group P compared to Group T (*p* < 0.001) (Table 2).

**Figure 1:**
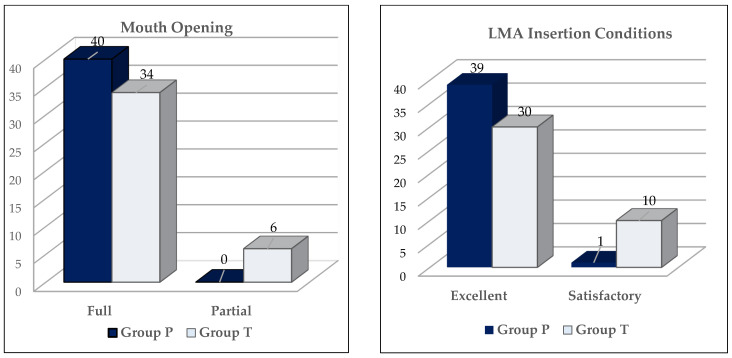
Laryngeal mask airway insertion condition: Assessment of insertion quality and mouth opening.

The rate of full mouth opening (*p* = 0.026) and excellent LMA insertion condition were significantly higher in Group P compared to Group T (*p* = 0.003) (Figure 1). Between Group P and Group T, there were no significant differences identified for LMA duration of use, ease of LMA insertion, acceptable LMA insertion condition (excellent and satisfactory LMA insertion conditions), movement, swallowing, coughing–retching rate, laryngospasm incidence, and the rate of successful LMA insertion on the first attempt (97.5% and 95%, in Groups P and T, respectively) (*p* > 0.05) (Table 3 and Figure 1). No more than two insertion attempts were required in either group.

Between Group P and Group T, no significant differences were identified among the basal HR, SAP, DAP, and MAP values (*p* > 0.05). When compared to values at baseline and 1 min before LMA insertion, HR, SAP, DAP, and MAP values decreased significantly after insertion in both groups (*p* < 0.05). In Group P, the HR, SAP, DAP, and MAP values 1 min before LMA insertion and at all time points after LMA insertion were significantly lower compared to Group T (*p* < 0.05) (Table 3).

The occurrence of hypotension or bradycardia was markedly higher in Group P compared to Group T (*p* < 0.05) (Table 4). No statistically meaningful differences were observed between Group P and Group T regarding the detection of blood on the LMA, sore throat, dysphonia, or dysphagia upon discharge from recovery (*p* > 0.05) (Table 4).

## 3. Discussion

According to the literature screening performed in our study and references we can access, this is the first study evaluating the effect of administration of remifentanil with thiopental on LMA insertion conditions and comparing with propofol. The results of our study show that the administration of thiopental after 2 μg·kg^−1^ remifentanil provided acceptable LMA insertion conditions (excellent and satisfactory LMA insertion conditions) at comparable levels to propofol administration, along with longer duration to eyelash reflex loss and LMA insertion duration, shorter apnoea duration, and more stable haemodynamics without increased hypotension or bradycardia risk.

Less stimulation occurs during laryngeal mask insertion compared to endotracheal intubation [15]. In this situation, lower doses of remifentanil may provide appropriate anaesthesia conditions [32]. Bouvet et al. reported that with 2.5 mg·kg^−1^ propofol, the dose of remifentanil required to obtain perfect muscle relaxant-free intubation conditions for 95% of patients was 4 μg·kg^−1^, while the remifentanil dose required to obtain excellent LMA insertion conditions for 95% of patients was 1.32 μg·kg^−1^. This is equivalent to nearly one-third of the dose required for tracheal intubation [25,27]. However, using the same study design with 5 mg·kg^−1^ thiopental, the remifentanil dose needed to achieve excellent intubation conditions without muscle relaxants in 95% of patients was reported as 7.8 µg·kg^−1^. [33] These three dose–response studies only included female patients. Previous studies have indicated that gender may influence the analgesic efficacy of opioids, and because men tend to require higher remifentanil effect-site concentrations (Ce) compared to women, appropriate dose adjustments should take gender into account when administering remifentanil for LMA placement [34]. Therefore, based on these considerations, our study determined that the selected remifentanil dose would remain below one-third of the amount necessary to achieve ideal intubation conditions without muscle relaxants in 95% of patients, and accordingly, 2 µg·kg^−1^ remifentanil was administered with 5 mg·kg^−1^ thiopental.

Opioids used as antitussives are known to inhibit the central mechanism of coughing and reduce pain-induced airway sensitivity in a dose-dependent manner [34]. Remifentanil demonstrates a fast onset of action and undergoes rapid breakdown by non-specific esterases in the blood and tissues, independent of organ function. Owing to its extremely brief duration of action, it enables swift emergence following prolonged ambulatory interventions, regardless of the total infusion time. Additionally, in situations requiring short-duration but intense narcotic effects like LMA insertion or tracheal intubation, it is the ideal opioid choice for outpatient interventions due to suppressing the hemodynamic response [23,25,35]. In previous reports, when administered with propofol, it was superior compared to alfentanil and fentanyl in terms of achieving 95–100% excellent LMA insertion conditions; however, there was 5–30% variation in ephedrine requirements along with significant falls in mean HR and MAP values independent of dose following induction [25,26,36,37]. Thiopental causes less hypotension compared to propofol. However, with the administration of fentanyl [8] or alfentanil [6] before induction with thiopental, there were higher incidences of coughing and laryngospasm along with lower rates of excellent and/or acceptable LMA insertions conditions compared to propofol and opioid administration. In our study, administration of remifentanil before induction with thiopental caused statistically significantly lower full mouth opening (85% vs. 100%) and excellent laryngeal mask insertion conditions (75% vs. 97.5%) (*p* < 0.01), along with similar rates of coughing or retching (2.5% vs. 0%), swallowing (7.5% vs. 2.5%), movement (2.5% vs. 0%), ease of LMA insertion (95% vs. 100%), number of patients with two attempts (5% vs. 2.5%), and acceptable laryngeal mask insertion conditions (100% vs. 100%) compared to propofol administration. No instances of laryngospasm were recorded in any patient in either group. The current findings show that administration of remifentanil, with a more potent effect and more suppression of reflex responses compared to other opioids, along with thiopental, which has less laryngeal response and less depressant effect on upper airway reflexes compared to induction with propofol, suppressed airway reflexes and provided LMA insertion success at similar rates to propofol administration.

According to the sources we accessed in the literature, although we did not use the same evaluation scales, three different induction techniques have been defined in addition to 5 mg·kg^−1^ thiopental, which is reported to provide comparable levels of excellent or acceptable LMA placement conditions (above 95%) when combined with 2.5 mg·kg^−1^ propofol following 1 µg·kg^−1^ fentanyl. Firstly, Seavell et al. [11] reported that 40 mg topical lidocaine sprayed on the posterior pharyngeal wall before IV 1 μg·kg^−1^ fentanyl before induction with thiopental provided higher hemodynamic stability and shorter apnoea duration compared to propofol administration. However, when Group T is compared with topical lidocaine administration, decreased incidences of coughing/retching (7.5% vs. 30.4%) were noted, and no patients developed laryngospasm (0% vs. 4.3%), indicating that remifentanil administration suppressed upper airway reflexes more effectively. In our study, SAP, DAP and HR were reduced by 14%, 16%, and 9%, respectively, compared to basal values after anaesthesia induction, while it was reported that with topical lidocaine administration, SAP and DAP reduced by 12% and 9%, while HR increased by 7%. In both administrations, hemodynamic depression was lower compared to propofol, with hypotension and bradycardia not observed. In our study with the administration of remifentanil with shorter effect duration instead of fentanyl, the longer mean apnoea duration (96 s vs. 244 s) may be associated with our premedication practice (midazolam). Though it may be a suitable alternative in terms of cost, short apnoea duration and stable hemodynamics, in terms of administration, the use of a topical anaesthesia for a short-duration anaesthesia procedure especially may increase the risk of vomiting with high probability during waking from anaesthesia or at surficial anaesthesia levels or aspiration linked to regurgitation caused by weakening or disappearance of the protective laryngeal reflex against aspiration.

Secondly, Koh et al. [10] reported that priming with a low dose of atracurium (0.1 mg·kg^−1^) before induction with thiopental resulted in a reduced incidence of laryngospasm compared to propofol (0% vs. 13.3%) while maintaining comparable acceptable LMA insertion rates. However, in Group T, when compared to priming administration, equivalent rates of acceptable LMA insertion conditions (100%, 100%) were observed, along with a lower incidence of laryngospasm (0% vs. 13.3%) and decreased occurrences of coughing/retching (2.5%, 13.3%), indicating that remifentanil administration more effectively suppressed upper airway reflexes. In our study, the longer mean apnoea duration (180 s versus 244 s, respectively) could be attributed to the greater proportion of male patients in the priming group, but we believe that the comparison may not be appropriate since the starting point for calculating apnoea duration was not clearly defined. Although the priming technique may be a suitable alternative for shorter apnoea duration, it can cause many unpleasant symptoms such as double vision, weakness, hypoventilation, and aspiration of gastric contents. Particularly in elderly patients, it may lead to greater decreases in pulmonary function, increased desaturation, and prolonged apnoea duration.

Thirdly, Bapat et al. [13] reported that the administration of 0.1 mg·kg^−1^ midazolam prior to induction using thiopental yielded comparable proportions of excellent and/or acceptable LMA insertion conditions (96%, 92%) relative to propofol use. However, when Group T is compared with the results from Bapat et al. though our full mouth opening, the rate slightly reduced (85% vs. 94%), there were fewer occurrences of coughing/retching (2.5% vs. 10%), movement (2.5% vs. 10%), and swallowing (5% vs. 14%), and laryngospasm was not observed in any subject (0% vs. 6%), suggesting—in a similar manner—that remifentanil administration suppressed upper airway reflexes at higher rates. Though it was reported that discharge criteria were not affected, midazolam administration caused high sedation scores in the postoperative first hour, and this may restrict its use for LMA insertion chosen for short-duration or outpatient surgery due to lengthening the emergence time. Given the comparable incidence of excellent or satisfactory LMA insertion conditions observed in our study, we consider the pre-induction delivery of 2 μg·kg^−1^ remifentanil to be a suitable option for brief and/or ambulatory surgical procedures, owing to its greater efficacy in suppressing airway reflexes without a notable increase in apnoea duration when compared to the three referenced regimens.

As propofol suppresses the baroreflex response in addition to its myocardial depression and vasodilator effects, the increase in HR is smaller despite a significant decrease in arterial pressure after induction [24,25,26,28]. Remifentanil has been linked to pronounced cardiovascular depressive events, including reductions in HR and/or arterial pressure, occurring more frequently than with other opioids [24,27,28,36,38]. Furthermore, bradyarrhythmia, which is commonly observed with remifentanil in clinical settings, has been attributed to a transient predominance of parasympathetic over sympathetic tone [38]. Consequently, the likelihood of bradycardia and hypotension is elevated when remifentanil is co-administered with propofol. The reduction in MAP and HR is generally manageable in individuals who are physiologically stable and adequately hydrated; however, it can pose a substantial risk to older adults or those with underlying cardiovascular or cerebrovascular pathology [24,25,26]. McCollum and Dundee [39] either 2.0 or 2.5 mg·kg^−1^ propofol to unpremedicated patients and noted it resulted in more pronounced reductions in mean systolic pressure (15%, 17%, and 10%, respectively) and hypotension compared to 5 mg·kg^−1^ thiopental. In an investigation evaluating the impact of remifentanil (0.5 µg·kg^−1^.min^−1^ infusion initiated 5 min prior to induction) in conjunction with propofol (2 mg·kg^−1^), thiopental (5 mg·kg^−1^), and etomidate (0.3 mg·kg^−1^), Wilhelm et al. [36] observed that MAP decreased by 26% with the remifentanil–propofol combination and by 18% with remifentanil–thiopental. As a result, with respect to the cardiodepressant properties of induction agents, they noted that this effect remained evident when used in combination with remifentanil, and the extent of hemodynamic suppression was notably intensified [2,24,36,39]. A study examining the influence of remifentanil in modulating cardiovascular reactions during orotracheal intubation by Thompson et al. [28] administered a 1 µg·kg^−1^ bolus followed by a 0.5 µg·kg^−1^ remifentanil infusion in the absence of any vagolytic agent. Five out of ten patients had bradycardia or hypotension or both observed, with this observed in 1 out of 10 patients in the group receiving 200 μg glycopyrrolate. Similarly, Klemola and colleagues [29] reported that the use of an anticholinergic premedication is advisable when high doses of remifentanil (3 or 4 µg·kg^−1^) are given in advance of induction with propofol, noting that this approach supported the maintenance of heart rate stability observed in their study. Nevertheless, with lower remifentanil doses, the administration of a vagolytic agent is often unnecessary. Stevens and Wheatley [40] infused 0.9% physiological saline at a volume of 7 mL·kg^−1^ prior to initiating anaesthesia with 2 mg·kg^−1^ propofol and 1–4 µg·kg^−1^ remifentanil, and documented that no instances of bradycardia or hypotension occurred in any subject. In agreement with previous reports, in our study, SAP, DAP, MAP, and HR values showed a marked reduction with propofol administration after anaesthesia induction compared to thiopental (20%, 22%, 21%, 15% vs. 14%, 16%, 15%, 9% in Groups P and T, respectively), and this decrease was also evident in both groups relative to baseline, continuing to decline throughout the study period. Following LMA insertion, increases in blood pressure and heart rate were not observed in both groups [9,25,27,29,30,31,33,35,41,42]. We did not use anticholinergic premedication due to the low dose of remifentanil administered. However, the occurrence rate of hypotension or bradycardia was higher with propofol compared to thiopental (15% vs. 0%). There are two tracheal intubation studies not using muscle relaxant, administering similar induction protocols to our study. Firstly, Siddik et al. [24] administered 1.5 mg·kg^−1^ lidocaine, 2 μg·kg^−1^ remifentanil, then 2.0 mg·kg^−1^ propofol, or 5 mg·kg^−1^ thiopental. After induction, propofol administration caused a significant reduction in HR compared to basal values, while there was no significant change in the thiopental group (13.8% and 0.5% in Groups P and T, respectively). Though more with propofol, both groups had a significant reduction in MAP compared to basal values (27.4% and 21.8% in Groups P and T, respectively) and higher rates of hypotension were reported in the propofol group (23.6% and 2.6% in Groups P and T, respectively). Propofol administration at a lower dose compared to our study caused higher rates of hemodynamic depression in both groups. This may be associated with the lower volume of fluid administration before induction (5 mL·kg^−1^ ringer lactate), shorter duration of administration for remifentanil and induction agents (30 s and 20 s, respectively), additional administration of 1.5 mg·kg^−1^ lidocaine in both groups and the higher rate of women patients. Erhan et al. [41] administered 0.01 mg·kg^−1^ atropine and 3 μg·kg^−1^ remifentanil, then 2.0 mg·kg^−1^ propofol or 6 mg·kg^−1^ thiopental for induction. Similarly, both groups had significant falls in MAP after induction, while there was a significant reduction in HR compared to basal values in the propofol group, there was no significant change in the thiopental group. After intubation, contrary to a significant increase in hemodynamic parameters in the thiopental group, there was a significant reduction with propofol administration, while no patient was administered ephedrine or atropine. Though there was administration of remifentanil at a higher dose compared to our study, hypotension and/or bradycardia were not reported in both groups. This may be related to the low dose of propofol administration, administration of remifentanil in shorter duration with longer administration of induction agents (10 s and 90 s, respectively), use of tracheal intubation with higher stimulus intensity, administration of additional atropine in both groups, and the study only including young men. When our study findings are evaluated alongside previous reports, it is evident that pre-induction hydration allows the suppression of the hemodynamic response during LMA placement without significant cardiovascular depression in thiopental-remifentanil administration. However, in propofol–remifentanil administration, the use of premedication with an anticholinergic agent in the absence of contraindications, or the administration of lower doses of remifentanil and/or propofol along with pre-induction hydration, may provide more stable hemodynamics by reducing the risk of hypotension or bradycardia.

Insertion of LMA requires anaesthesia depth that is adequate to prevent airway reflexes. The doses of remifentanil, propofol, and thiopental necessary to achieve sufficient anaesthesia vary among individuals. Therefore, using a fixed dose can lead to airway complications and cardiorespiratory depression associated with either insufficient or excessive anaesthesia [34,43]. The use of a bispectral monitor is a clinical alternative to evaluate anaesthesia depth during airway manipulation. BIS monitoring has been shown to allow titration of anaesthetic doses according to anaesthesia level, thus decreasing the frequency of hemodynamic disturbances and enhancing recovery outcomes [44]. We completed LMA insertion when sufficient mouth relaxation was present and when BIS was under 40, with additional dose given if needed. As a result, with standard dose administration, we think we prevented different LMA insertion conditions that may emerge linked to variable LMA insertion timing related to the inserter and anaesthesia depth varying as a result of dose requirements linked to individual variables.

Wilhelm et al. [36] reported that the time required for the loss of the eyelash reflex was markedly reduced when remifentanil was administered in combination with propofol, thiopental, and etomidate compared to fentanyl administration; hence, the quality of induction improved. In our study, in alignment with the findings of Afridi et al. [45], and using a similar dose and protocol, the time to loss of the eyelash reflex was considerably lower with propofol administration (23.8 s vs. 41.7 s, respectively) compared to thiopental (30.1 s vs. 51.1 s, respectively). In both groups, the average time to achieve the loss of the eyelash reflex was shorter, which may be attributed to our additional administration of remifentanil [7,45]. Under these conditions, consistent with the observations by Wilhelm et al. [36], it can be stated that both the duration and the quality of induction were enhanced with remifentanil administration.

The mean duration required for LMA insertion may be influenced by the clinician’s experience, the type of LMA used, the insertion technique applied, the method of time measurement, and the variability of induction agents across groups [46]. We recorded the duration from mouth opening until the first successful ventilation as the LMA insertion duration [46]. In accordance with previous studies, and reflecting the enhanced LMA insertion conditions observed in our research, the duration of LMA insertion was markedly reduced with propofol administration (mean 10.5 s) relative to thiopental administration (mean 12.6 s) [42,46].

Airway injuries are recognised complications of general anaesthesia (GA). Although the reported incidence varies widely, LMA use has been shown to result in lower rates of postoperative pharyngolaryngeal morbidity and dysphonia than tracheal intubation [47,48]. Throat pain, which may exacerbate postoperative morbidity and reduce patient satisfaction, is identified as the eighth most common adverse event in the postoperative period following GA [48]. The incidence of throat pain ranges from 24% to 90% after endotracheal intubation [49]. Following LMA use, the reported incidence of postoperative throat pain spans a broad range of 0–43% [2,47]. The occurrence of LMA-related pharyngeal morbidity is influenced by several factors. Trauma sustained during device placement is considered the primary cause of airway morbidity associated with LMA [2]. The incidence of throat pain related to LMA can be affected by multiple variables, including the depth of anaesthesia at insertion, use of neuromuscular blocking agents, insertion technique, clinician experience, number of attempts, oversized LMA, excessive cuff volume or pressure, duration of anaesthesia, type of postoperative analgesia, cuff pressure elevation due to N_2_O diffusion, and the use of heated humidifier exchangers in the circuit [47,48]. Chui et al. [50] performed a study assessing the impact of propofol used with low-dose mivacurium on LMA insertion conditions. They reported that administration of increasing doses of mivacurium in addition to propofol caused a reduction in adverse reaction incidence, an increase in LMA ease of insertion, along with a fall in throat pain incidence from 53% to 24%. Chia et al. [47] observed a reduced incidence of early pharyngeal morbidity (throat pain 24% vs. 13%, dysphagia 15% vs. 3% for thiopental and propofol at the second postoperative hour, respectively), including throat pain and dysphagia, attributed to the greater suppression of laryngeal reflexes by propofol compared to thiopental. In our study, no significant differences were observed between the propofol (10%, 2.5%) and thiopental (15%, 10%) groups with respect to throat pain and dysphagia. The incidences of dysphagia and throat pain in our findings align with previous reports, and we suggest that the absence of a significant difference between groups may be due to the comparable LMA insertion conditions and the similar number of insertion attempts.

A limitation of our study is that our findings are applicable only to patients aged 18–65 years in ASA classes I and II undergoing elective surgeries with premedication. In ASA III or IV patients, particularly individuals with advanced cardiac disease or those undergoing emergency procedures without premedication or in anxious patients, there may be altered hemodynamic tolerance to remifentanil administered at doses up to 2 µg·kg^−1^ in combination with propofol. Likewise, because the age-related changes in the pharmacokinetic and pharmacodynamic profiles of propofol, thiopental, and remifentanil can influence drug responses, our results are likely not generalisable to elderly patients [27]. The absence of a control group in our study can be regarded as a limitation. Nevertheless, the likelihood of airway trauma and inadequate ventilation could rise if pharyngolaryngeal reflexes are not adequately suppressed through the use of thiopental and propofol alone. We did not create a control group as we did not consider this to be ethical. We also share the view that another limitation is the absence of postoperative nausea and vomiting (PONV) assessment, which is clinically relevant—particularly considering the known association of thiopental with increased PONV. Although not included in our predefined outcomes, we agree that evaluating PONV represents an important perspective for future studies in this field.

## 4. Materials and Methods

### 4.1. Study Population

The study included 80 patients in ASA physiological classification groups I–II, aged 18–65 years, undergoing elective surgery, not requiring muscle relaxants, with operation duration less than 2 h and indications for LMA insertion. Cases with any neck and upper respiratory tract pathology, history and probability of difficult airway (Mallampati class 3–4, sternomental distance less than 12 cm, thyromental distance less than 6 cm, head extension below 90 degrees, and mouth opening less than 1.5 cm), who were morbidly obese (Body Mass Index (BMI) > 40 kg/m^2^), with history of lung disease, with allergy to the study drugs, with history of alcohol and drug dependence, history of chronic sedative and opioid use, throat pain, dysphagia or dysphonia were excluded from the study.

### 4.2. Study Design

This prospective, randomised trial adopted a parallel-group design. Patients were randomly assigned to receive 2.5 mg·kg^−1^ propofol or 5 mg·kg^−1^ thiopental with 2 μg·kg^−1^ remifentanil administered before induction (Groups P and Groups T, respectively, with *n* = 40 in each group). Randomisation was performed using a closed-envelope method to ensure the integrity of group allocation. The primary outcomes of this study were LMA insertion conditions, time to eyelash reflex loss, and time to successful LMA insertion, while the secondary outcomes included hemodynamic response, apnoea duration, pharyngolaryngeal morbidity, and incidence of complications.

### 4.3. Anaesthetic Management

Patients taken to the operating room had standard monitoring before anaesthesia induction [heart rate (HR), systolic arterial pressure (SAP), diastolic arterial pressure (DAP), mean arterial pressure (MAP), electrocardiography (ECG-derivation II), and peripheral oxygen saturation (SpO2)]. Anaesthesia depth was monitored with bispectral index monitoring [BIS-Vista™ (Aspect Medical Systems; Newton, MA, USA)] [26,33,34,42]. Patients had venous access in the back of the hand with 20 G cannula, and saline infusion of 7 mL·kg^−1^ was administered before induction [30,31,35,41]. Patients were randomly divided into 2 groups with the closed envelope method as 2 μg·kg^−1^ remifentanil followed by 2.5 mg·kg^−1^ propofol (Group P, *n* = 40) or 5 mg·kg^−1^ thiopental (Group T, *n* = 40). The induction sequence was completed using two previously prepared syringes. Syringe 1 contained 2 µg·kg^−1^ remifentanil, and the total of 50 mL volume was filled with saline. Syringe 2 used 20 mL injectors and contained 1% propofol (10 mg/mL) or 2% thiopental (20 mg/mL) concentration covered with opaque tape to prevent seeing the contents. As the study protocol was planned to be double blind, the coded test syringes were prepared by a nurse not participating in the study. As the anaesthesiologist monitoring LMA insertion and parameters was blind to the drug doses, injection of all syringes was performed by an assistant behind a cover [41,47]. In this situation, the drug preparation, administration, insertion of LMA and monitoring of parameters were performed by different people. In this way, it was ensured that the anaesthesiologist who inserted the LMA, assessed patient response, and monitored parameters was blind to the drugs administered [30,31,41]. Nearly three minutes before induction, all patients underwent preoxygenation with 6 L/min oxygen via face mask [34] and premedication with 0.03 mg/kg^−1^ IV midazolam (Dormicum^®^ ampoule, Roche Pharmaceuticals, Istanbul, Türkiye), followed by the administration of the study drugs [6,13]. Remifentanil with 2 μg·kg^−1^ dose was infused over 60 s with an infusion pump (Braun Infusomat^®^; Braun Melsungen Ko, Melsungen, Germany) [3,41]. Then, 30 s after remifentanil infusion began, the patients were given 0.25 mL·kg^−1^ fixed dose of hypnotic agent administered over 30 s for induction [8,10,22,42,47].

With eyelash reflex checks after induction, patients were ventilated with 100% O_2_ through a face mask [24,30]. The duration to loss of eyelash reflex was determined as the time from beginning administration of the induction agent to the time when eyelash reflex was lost, and this was recorded [7,26,36]. Ninety seconds after thiopental administration [24,30,41], when BIS values were under 40 [26] and sufficient mouth relaxation was provided, the size of the LMA was determined according to patient body weight as recommended in the manufacturer’s guidelines, and the same type of mask (Classic) was used for all patients in the study. The side of the LMA facing the oropharynx was lubricated with a water-soluble gel, the cuff was fully deflated, and the LMA was inserted with the standard method described by Brian by a single researcher with more than 3 years of experience [13,46,47]. After inserting the LMA, cuff pressure monitoring was performed to standardise postoperative pharyngeal morbidity (cuff pressure manometer, Rüsch, Kernen, Germany). The laryngeal mask cuff was inflated until the air in the cuff reached 20–30 cmH_2_O and was maintained below 40 cmH_2_O throughout the operation (cuff pressure manometer, Rüsch, Kernen, Germany) [2,51].

The duration to successful insertion (duration from opening of mouth to first successful ventilation) was recorded [46]. Criteria for successful insertion of the laryngeal mask were assessed as observation of square waves on capnogram, easy ventilation with the respiration balloon, observation of chest movements and no air leak with maximum 20 cmH_2_O positive pressure ventilation [46,52]. Anaesthesia maintenance was provided by a 50% O_2_/50% N_2_O mixture containing 1.5–2% sevoflurane [33]. Sevoflurane concentration was set so BIS values were between 40 and 60 [26]. If sufficient induction could not be provided in patients, if any movement was observed during the first attempt and if necessary to keep BIS values below 40, 0.125 mL·kg^−1^ equivalent additional doses of each study drug were administered and then 60 s later, LMA insertion was attempted a second time [26,47,50]. The number of attempts was recorded; however, LMA insertion conditions were only evaluated during the first attempt [4,46,50]. In cases where two attempts at placement failed (for reasons such as inability to achieve adequate ventilation, the presence of audible air leaks, and failure to resolve these issues by repositioning; hypercarbia [end-tidal CO_2_ > 45]; or hypoxemia [SpO_2_ dropping below 90% at any point during the study, or complete laryngospasm]), intubation was performed and the patients were excluded from the study [10,26,47,51,52]. For assessment of LMA insertion conditions, a 6-variable scale used in previous studies, including mouth opening, ease of insertion, swallowing, coughing/retching, laryngospasm and patient movements, was used [25,50,53]. The tolerance of patients to LMA insertion was assessed. LMA insertion conditions were evaluated in three groups; excellent when all criteria were excellent; satisfactory when all criteria were a mix of satisfactory and excellent; and poor when one or more criteria were poor.

In all two groups, SAP, DAP, MAP, HR, BIS, and SpO_2_ values were recorded at basal point, 1 min before LMA insertion and 1 min, 2 min, 3 min, 4 min and 5 min after insertion. The apnoea duration (duration between last spontaneous respiration after induction to the start of first spontaneous respiration) was recorded [26,42]. Five minutes before the end of operation, 100% O_2_ was begun. Before removing the LMA, the cuff pressure was measured again and recorded. In the presence of sufficient ventilation, the LMA was removed, and the duration of LMA use was recorded (duration between insertion and removal) [54]. After removing the laryngeal mask, the presence of blood was assessed as 1: no blood seen, 2: trace amounts and 3: significant amount of blood [55]. Waking patients were taken to the recovery unit with 100% oxygen. To determine the incidence and severity of pharyngolaryngeal complications, patients were assessed on discharge from the recovery unit for throat pain (constant pain, independent of swallowing) and difficulty swallowing (dysphagia: difficulty with swallowing provoked by drinking) by a single researcher blind to the group and not included in the anaesthesia process. Patients were questioned specifically about the presence/absence of symptoms in the postoperative period. Throat pain was assessed as follows: 0 points = no complaints, 1 point = mild throat pain, 2 points = moderate throat pain and 4 points = severe throat pain [49,54]. If hypotension occurred in the peroperative period (<30% reduction in MAP compared to basal values), 6 mg ephedrine (Ephedrine, Haver, İstanbul, Türkiye) was administered. Bradycardia was defined as HR less than 50 beats/min and 0.5 mg IV atropine (Atropine sulphate, Haver, İstanbul, Türkiye) was administered [35,41] (Table 5).

### 4.4. Ethical Statement

This study was conducted in compliance with the Declaration of Helsinki, approved by the Okmeydani Education and Research Hospital Medication Research Local Ethics Committee (decision number: 132), and registered with the Australian New Zealand Clinical Trials Registry (ACTRN12625000275460). All patients provided written informed consent prior to participation in the study, acknowledging their understanding of the study’s purpose, procedures, and potential risks.

### 4.5. Statistical Analysis

Descriptive statistics (mean, standard deviation, median, minimum, maximum) were used to describe continuous variables. The distribution of categorical variables was presented using frequencies and percentages. The normality of the data distribution was assessed using the Kolmogorov-Smirnov test. The comparison of two independent continuous variables that did not follow a normal distribution was analysed using the Mann–Whitney U test. Paired sample *t* test was used in the comparison between two dependent continuous variables. The relationship between categorical variables was examined using the Chi-Square test (or Pearson/Fisher’s Exact test, where appropriate). The effect of time in groups was examined with a Repeated Measures ANOVA test. A *p*-value of less than 0.05 was considered statistically significant. The analyses were performed using MedCalc Statistical Software version 12.7.7 (MedCalc Software bvba, Ostend, Belgium; http://www.medcalc.org; 2013).

### 4.6. Power Analysis

Posteriori power analysis was conducted using the G Power 3.1.9.6 software. When the distributions for Excellent LMA Insertion conditions in the P and T groups were set to 0.75 and 0.975, respectively, and with a Type I error of 0.05 and a two-tailed hypothesis, the test power was calculated to be 86.6%.

## 5. Conclusions

This prospective, randomised, and double-blind clinical study demonstrated that both propofol–remifentanil and thiopental–remifentanil combinations can be effectively used to achieve optimal conditions for classic laryngeal mask airway (LMA) insertion without the use of muscle relaxants. While both groups provided satisfactory insertion conditions, the thiopental-remifentanil combination was associated with significantly more stable hemodynamic responses and a shorter apnoea duration. In contrast, the propofol-remifentanil group showed a higher rate of full mouth opening and excellent LMA insertion conditions, along with a slightly faster onset of unconsciousness; however, this was accompanied by a higher incidence of hypotension and bradycardia. These findings suggest that thiopental, when used in combination with remifentanil, remains a clinically relevant alternative to propofol, particularly in outpatient surgical procedures or settings where cardiovascular stability is a priority.

In conclusion, thiopental-remifentanil appears to be a safe, effective, and hemodynamically advantageous option for LMA insertion in suitable patients. Further studies involving broader patient populations, including the elderly and high-risk groups, may help to confirm its role in modern anaesthetic practice.

## Figures and Tables

**Table 1:** Demographic data: Age, sex, and weight distribution of patients in Group P (propofol–remifentanil) and Group T (thiopental–remifentanil).

			Group P (*n* = 40)	Group T (*n* = 40)	*p*-Value
Age(year)		Mean ± SD	44.3 ± 11.1	41.6 ± 14	0.338 ^1^
		Median (Min.–Max.)	44.5 (19–63)	44.5 (18–65)	
Sex	Female	*n* (%)	14 (35.0)	18 (45.0)	0.361 ^2^
	Male	*n* (%)	26 (65.0)	22 (55.0)	
Weight (kg)		Mean ± SD	73.3 ± 12.7	72.4 ± 11.3	0.731 ^1^
		Median (Min.–Max.)	72 (50–102)	73.5 (48–93)	

^1^ Independent sample *t* test; ^2^ Pearson Chi-Square.

**Table 2:** Induction Parameters: Comparison of eyelash reflex loss, apnoea time, LMA insertion duration, and usage time between the two groups.

Variables		Group P (*n* = 40)	Group T (*n* = 40)	*p*-Value
Duration to loss of eyelash reflex (s)	Mean ± SD	23.8 ± 2.4	30.1 ± 2.3	<0.001 ^2^
	Median (Min.–Max.)	24 (20–29)	30.5 (25–34)	
Apnoea duration (s)	Mean ± SD	376.3 ± 42	244.1 ± 45.9	<0.001 ^1^
	Median (Min.–Max.)	377.5 (296–440)	240 (160–370)	
LMA insertion duration (s)	Mean ± SD	10.5 ± 1.4	12.6 ± 1.7	<0.001 ^2^
	Median (Min.–Max.)	10 (9–14)	13 (10–16)	
LMA use duration (min)	Mean ± SD	50.4 ± 16.9	53.4 ± 17.9	0.272 ^2^
	Median (Min.–Max.)	44 (35–104)	49 (32–111)	

^1^ Independent sample *t* test, ^2^ Mann–Whitney U-test.

**Table 3:** Haemodynamic parameters: Heart rate, systolic, diastolic, and mean arterial pressures recorded at baseline, 1 min pre-insertion, and at 1, 2, 3, 4, and 5 min after insertion.

	Group P (*n* = 40)		Group T (*n* = 40)		*p*-Value
	Mean ± SD	Median (Min.–Max.)	Mean ± SD	Median (Min.–Max.)	
Heart Rate (bpm)					
Baseline	76.2 ± 11.1	76 (56–106) ^b^	77.2 ± 13.7	76 (56–105) ^b^	0.714 ^1^
1 min Before	65.1 ± 9.8	64 (50–89) ^a^	70.7 ± 12.5	70 (52–97) ^a^	0.028 ^1^
1 min After	63.7 ± 9.6	62 (50–87) ^a,b^	69.1 ± 11.5	68 (51–93) ^a,b^	0.026 ^1^
2 min	62.1 ± 9.5	62 (49–85) ^a,b^	67.6 ± 11	67 (51–90) ^a,b^	0.024 ^2^
3 min	59.7 ± 8.6	59 (49–81) ^a,b^	65.2 ± 10.3	63.5 (50–88) ^a,b^	0.014 ^2^
4 min	59.2 ± 8.3	59 (48–80) ^a,b^	64.7 ± 10.5	63 (50–86) ^a,b^	0.017 ^2^
5 min	58.6 ± 8.5	58 (48–82) ^a,b^	64.1 ± 10.4	62.5 (49–87) ^a,b^	0.014 ^2^
Systolic arterial pressure (mmHg)					
Baseline	131.7 ± 17.1	132 (95–165)	130.9 ± 20.7	129.5 (94–170)	0.856 ^1^
1 min Before	105.3 ± 12	106 (78–125) ^a^	112.8 ± 16.6	113 (82–144) ^a^	0.023 ^1^
1 min After	104.3 ± 12	106 (77–123) ^a,b^	111.6 ± 16.2	111 (82–142) ^a,b^	0.026 ^1^
2 min	103.5 ± 12.1	105.5 (76–121) ^a,b^	110.8 ± 16.6	110 (80–141) ^a,b^	0.027 ^1^
3 min	99.8 ± 10.5	101 (75–117) ^a,b^	107.2 ± 15.3	106.5 (80–138) ^a,b^	0.014 ^1^
4 min	98.2 ± 10.1	99 (75–115) ^a,b^	105.5 ± 14.7	104 (82–135) ^a,b^	0.012 ^1^
5 min	97.4 ± 9.9	98.5 (74–115) ^a,b^	104.8 ± 14.8	103.5 (81–136) ^a,b^	0.011 ^1^
Diastolic arterial pressure (mmHg)					
Baseline	77 ± 11	76 (56–98) ^b^	78.4 ± 13.6	78 (56–106) ^b^	0.607 ^1^
1 min Before	61.2 ± 8.4	61.5 (45–78) ^a^	65.9 ± 11.1	65 (49–92) ^a,b^	0.034 ^1^
1 min After	60.7 ± 8	60.5 (47–75) ^a,b^	67.5 ± 11.4	67 (48–90) ^a,b^	0.003 ^1^
2 min	59.8 ± 7.7	59.5 (46–74) ^a,b^	65.3 ± 10.9	64.5 (48–90) ^a,b^	0.011 ^1^
3 min	58 ± 7.3	57 (46–71) ^a,b^	63.3 ± 10.4	62.5 (47–88) ^a,b^	0.010 ^1^
4 min	56.5 ± 6.9	55.5 (46–70) ^a,b^	62.4 ± 10.5	62 (47–88) ^a,b^	0.004 ^1^
5 min	55.8 ± 7.3	54.5 (45–70) ^a,b^	61.6 ± 10.6	61 (46–87) ^a,b^	0.017 ^2^
Mean arterial pressure (mmHg)					
Baseline	95.2 ± 11.6	94.5 (70–120)	95.9 ± 13.2	95 (69–125)	0.808 ^1^
1 min Before	75.9 ± 8.7	75.5 (57–92) ^a^	81.5 ± 10.5	81 (61–109) ^a^	0.011 ^1^
1 min After	75.3 ± 8.4	74 (57–90) ^a^	82.3 ± 10.6	82 (60–107) ^a,b^	0.002 ^1^
2 min	74.3 ± 8.3	74 (56–89) ^a^	80.4 ± 10.5	80 (60–107) ^a,b^	0.005 ^1^
3 min	72 ± 7.5	72 (56–86) ^a,b^	77.9 ± 10	77.5 (59–105) ^a,b^	0.004 ^1^
4 min	70.4 ± 7	71 (56–83) ^a,b^	76.8 ± 9.9	76.5 (59–104) ^a,b^	0.001 ^1^
5 min	69.6 ± 7.2	71 (55–82) ^a,b^	76 ± 10	76.5 (58–103) ^a,b^	0.001 ^1^

^1^ Student *t* test. ^2^ Mann–Whitney U-test, ^a^ differs significantly from baseline values (Wilcoxon Signed Rank test *p* < 0.001), ^b^ significantly different from the values of 1 min ago (Wilcoxon Signed Rank test *p* < 0.001).

**Table 4:** Postoperative complication: incidence of sore throat, bleeding, hypotension, bradycardia, and dysphagia in recovery.

		Group P (*n* = 40)		Group T (*n* = 40)		*p*-Value
Presence of blood	1	38	95%	37	92.5%	1.000 ^2^
	2	2	5%	2	5%	
	3	0	0%	1	2.5%	
Recovery sore throat	0	36	90%	34	85	0.499 ^1^
	1	3	7.5%	4	10%	
	2	1	2.5%	2	5%	
	3	0	0%	0	0%	
	4	0	0%	0	0%	
Complications	No	34	85%	40	100%	0.026 ^2^
	Bradycardia	2	5%	0	0%	
	Hypotension	3	7.5%	0	2.5%	
	Hypotension and Bradycardia	1	2.5%	0	0%	
Recovery dysphagia	No	39	97.5%	36	90%	0.359 ^2^
	Yes	1	2.5%	4	10%	

^1^ Pearson Chi Square, ^2^ Fisher’s Exact test. Comparisons were made between first and other categories (together).

**Table 5:** LMA insertion tolerance assessment scale: Scoring criteria used to evaluate insertion conditions based on mouth opening, ease of insertion, patient responses, and airway reflexes.

Variables	Ease of LMA Insertion		
	Excellent	Satisfactory	Poor
Mouth opening	Full	Partial	None
Ease of LMA insertion	Easy	Difficult	Impossible
	Patient response		
Swallowing	None	Mild	Pronounced
Coughing and retching	None	Mild	Pronounced
Head and body movements	None	Mild	Pronounced
Laryngospasm	None	Partial	Full

LMA insertion conditions: excellent, all responses are excellent; satisfactory, all responses are excellent or satisfactory; poor, the presence of one or more.

## Data Availability

Data is contained within the article.
